# New Ternary Blend Strategy Based on a Vertically Self‐Assembled Passivation Layer Enabling Efficient and Photostable Inverted Organic Solar Cells

**DOI:** 10.1002/advs.202206802

**Published:** 2023-04-25

**Authors:** Soyeong Jeong, Aniket Rana, Ju‐Hyeon Kim, Deping Qian, Kiyoung Park, Jun‐Ho Jang, Joel Luke, Sooncheol Kwon, Jehan Kim, Pabitra Shakya Tuladhar, Ji‐Seon Kim, Kwanghee Lee, James R. Durrant, Hongkyu Kang

**Affiliations:** ^1^ Department of Chemistry and Centre for Processable Electronics Imperial College London White City Campus London W12 0BZ UK; ^2^ School of Materials Science and Engineering Gwangju Institute of Science and Technology (GIST) Gwangju 61005 Republic of Korea; ^3^ Heeger Center for Advanced Materials (HCAM) Gwangju Institute of Science and Technology (GIST) Gwangju 61005 Republic of Korea; ^4^ Department of Physics and Centre for Processable Electronics Imperial College London London SW7 2AZ UK; ^5^ Department of Energy and Materials Engineering Dongguk University Seoul 04620 Republic of Korea; ^6^ Pohang Accelerator Laboratory (PAL) Pohang University of Science and Technology (POSTECH) Pohang 37673 Republic of Korea; ^7^ Research Institute for Solar and Sustainable Energies (RISE) Gwangju 61005 Republic of Korea

**Keywords:** inverted organic solar cells, nonfullerene acceptor, photostability, self‐assembled monolayers

## Abstract

Herein, a new ternary strategy to fabricate efficient and photostable inverted organic photovoltaics (OPVs) is introduced by combining a bulk heterojunction (BHJ) blend and a fullerene self‐assembled monolayer (C_60_‐SAM). Time‐of‐flight secondary‐ion mass spectrometry ‐ analysis reveals that the ternary blend is vertically phase separated with the C_60_‐SAM at the bottom and the BHJ on top. The average power conversion efficiency ‐ of OPVs based on the ternary system is improved from 14.9% to 15.6% by C_60_‐SAM addition, mostly due to increased current density (*J*_sc_) and fill factor ‐. It is found that the C_60_‐SAM encourages the BHJ to make more face‐on molecular orientation because grazing incidence wide‐angle X‐ray scattering ‐ data show an increased face‐on/edge‐on orientation ratio in the ternary blend. Light‐intensity dependent *J*_sc_ data and charge carrier lifetime analysis indicate suppressed bimolecular recombination and a longer charge carrier lifetime in the ternary system, resulting in the enhancement of OPV performance. Moreover, it is demonstrated that device photostability in the ternary blend is enhanced due to the vertically self‐assembled C_60_‐SAM that successfully passivates the ZnO surface and protects BHJ layer from the UV‐induced photocatalytic reactions of the ZnO. These results suggest a new perspective to improve both performance and photostability of OPVs using a facial ternary method.

## Graphical abstract

A new ternary system is introduced that can form a vertically self‐assembled passivation layer on the interface between photoactive layer and ZnO layer in n‐i‐p device architecture. This system not only improves photostability against UV light but also increases in *J*_sc_ and fill factor ‐ by encouraging to make a favorable molecular orientation of photoactive materials from edge‐on to face‐on orientation.

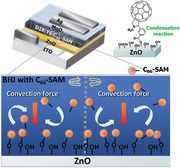

## 1 Introduction

Organic photovoltaics (OPVs) are attracting significant research attention due to their light weight, flexibility, and solution processability and therefore have their promising potential for future solar applications.^[^ ^1^, ^2^, ^3^ ^]^ Intensive research efforts have been devoted to develop donor and acceptor materials in order to improve power conversion efficiency (PCE),^[^ ^3^, ^4^, ^5^, ^6^ ^]^ with the development of Y6 and its derivatives as nonfullerene acceptors (NFAs) yielding efficiencies of over 15%.^[^ ^7^ ^]^ Moreover, with the further molecular engineering of donor polymers such as D18, remarkable PCEs based over 18% have been achieved by Li et al. for on D18:Y6 devices.^[^ ^8^ ^]^ Among other strategies to improve PCEs, ternary blend systems, which commonly consist of one donor and two acceptors or two donors and one accepter, are making further progress towards the highest OPV performance.^[^ ^2^, ^9^, ^10^ ^]^ Complementary effects of enhanced light absorption and morphology in such ternary systems have allowed improvements in device performance, with Liu et al. recently reporting a ternary OPV comprising two donors and one acceptor with the highest PCE of 19.3%.^[^ ^11^ ^]^ These high performance devices bring the OPV field closer to large scale commercialization. However, for such commercialization, some stability issues remain to be addressed, including excessive phase separation under thermal stress, photoinduced degradation of photoactive layers, deformation by mechanical stress, and interfacial instability induced by defect sites on charge collection layers.^[^ ^12^, ^13^, ^14^ ^]^ . Although the degradation mechanisms of polymer:fullerene‐based OPVs, triggered by factors such as oxygen, heat, and light, have been extensively studied during the past two decades, the stability studies for the state‐of‐the‐art NFA‐based OPVs have not progressed as rapidly because most research efforts have focused on enhancing the PCEs of the devices.^[^ ^15^, ^16^, ^17^, ^18^, ^19^ ^]^

From a structural point of view, the inverted n‐i‐p architecture has been normally considered as more stable compared to conventional p‐i‐n devices for fullerene‐based OPVs, due to their use of high work function metals as a stable top anode.^[^ ^20^, ^21^, ^22^ ^]^ For the high performance NFA‐based OPVs, however, this structure raises new stability issues at the interfaces between the photoactive layer and charge collection layers. Zinc oxide (ZnO) has been widely utilized as an electron transport layer in the inverted architecture owing to its high transparency in the visible region, high electron mobility, and high ambient stability. However, the UV‐light induced photocatalytic reactivity of the ZnO can be particularly destructive for some NFAs, especially those having end groups such as indandione or rhodanine in their molecular structure.^[^ ^23^ ^]^ Because these end groups are connected with core by a nonaromatic double bond, they can be easily attacked by the active radicals induced from photocatalytic reaction of ZnO.^[^ ^24^ ^]^ It has been demonstrated that photobleaching rapidly occurs in the NFAs such as IT‐4F, ITIC, and Y6 deposited as films on ZnO under UV illumination, so called “UV‐induced dimerization process.”^[^ ^23^, ^24^ ^]^ A deeper investigation of the ITIC reveals that the ITIC molecule can occur dimerization. These UV‐induced decomposition or dimerization processes can causes an absorption loss and increased charge trapping, resulting in increased charge recombination and shunt formation. Several surface modifiers, such as amine group‐functionalized perylene diiminde (PDIN),^[^ ^25^ ^]^ polyethyleneimine (PEI),^[^ ^26^ ^]^ and fullerene‐based self‐assembled monolayer (C_60_‐SAM),^[^ ^27^, ^28^, ^29^, ^30^ ^]^ have been introduced at the interface as a passivation layer to protect the photoactive layer from this harmful influence of ZnO. However, inserting surface modifiers in between ZnO and bulk heterojunction (BHJ) induces the complexity in fabrication of the OPVs, thereby increasing the potential cost of the OPV manufacturing. For instance, to fabricate the ZnO/C_60_‐SAM film, it is required to additionally coat the C_60_‐SAM layer onto the ZnO layer and then perform an extra rinsing process with a clean solvent for creating a C_60_‐SAM monolayer and preventing C_60_‐SAM aggregation. As a result, incorporating two additional processes could lead to considerable losses in terms of both time and cost for the OPV mass production.

In this work, we demonstrate a new ternary system that is able to form a vertically self‐assembled passivation layer on the interface between photoactive layer and charge collection layer for improving both device performance and photostability. Because of the self‐assembly characteristic of C_60_‐SAM, the new ternary system can offer a processing advantage that reduces the additional coating and rinsing processes for forming the C_60_‐SAM monolayer. The ternary blend consists of a BHJ composing D18 as a polymer donor and Y6 as NFA and a self‐assembled monolayer (C_60_‐SAM). The C_60_‐SAM can be vertically self‐assembled at the surface of ZnO layer by inducing a condensation reaction between the carboxylic acid end group of C_60_‐SAM and hydroxyl groups on the surface of ZnO, resulting in a pseudo C_60_‐SAM/BHJ bilayer. The self‐assembled C_60_‐SAM layer functions as a passivation layer that slows down the photoinduced degradation of the devices by suppressing photobleaching of BHJ against UV light. Moreover, the ternary OPV device shows improved device efficiency, mainly due to increased *J*sc and fill factor (FF). Grazing incidence wide‐angle X‐ray scattering (GIWAXS) and charge carrier lifetime analyses reveal that the C_60_‐SAM encourages a face‐on orientation of the BHJ, improving charge carrier lifetime and mobility.

## 2 Results and Discussion

**Figure 1:**
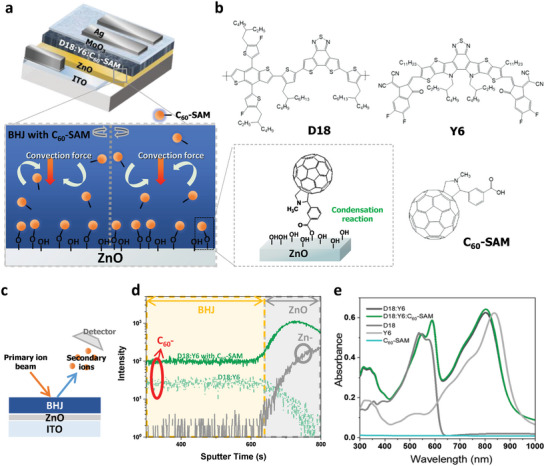
a) Schematic device structure of organic photovoltaics (OPV) devices and vertical separation in the D18:Y6:C_60_‐SAM solution. b) Molecular structures of photoactive materials (D18 and Y6) and an additive (C_60_‐SAM) used in this study. c) Scheme of time‐of‐flight secondary‐ion mass spectrometry (ToF‐SIMS) measurement and d) depth profiles of ZnO/D18:Y6 films without and with C_60_‐SAM additive. e) Absorption spectra of the neat D18, Y6, C_60_‐SAM, D18:Y6, and D18:Y6:C_60_‐SAM films.

**Figure** **1**a,b illustrates the inverted ITO/ZnO/BHJ/MoO_3_/Ag device structure and the molecular structures of the materials (D18, Y6 and C_60_‐SAM) used in this study. The energy levels of the materials are presented in Figure S1 (Supporting Information). The C_60_‐SAM was independently dissolved in isopropyl alcohol (IPA) at concentrations of 0.5, 1, 2, and 5 wt%, and then 0.5 vol% of these solutions was added to the BHJ solution. Because of the hydrophilic functional groups and self‐assembly characteristic of the C_60_‐SAM, the BHJ:C_60_‐SAM solution is expected to spontaneously induce vertical phase separation and to form a BHJ/C_60_‐SAM bilayer. When the BHJ:C_60_‐SAM solutions are spin‐coated on the ZnO, a strong centrifugal force induces a convection flow in the solution that can provide a sufficient driving force to move C_60_‐SAM onto the ZnO surface during film formation.^[^ ^31^ ^]^ Because the carboxylic acid groups contained in the C_60_‐SAM can undergo a condensation reaction with surface hydroxyl groups on ZnO, the C_60_‐SAM has the potential to self‐assemble on the ZnO surface (Figure 1a).^[^ ^27^, ^30^ ^]^

To confirm our expectation, we first performed time‐of‐flight secondary‐ion mass spectrometry (ToF–SIMS) to obtain vertical profiles of the atoms distribution in ITO/ZnO/D18:Y6 and ITO/ZnO/D18:Y6:C_60_‐SAM structures. As shown in the illustration for ToF–SIMS analysis in Figure 1c, secondary ions were detected starting from the top to bottom of the films. In the resulting depth profile (Figure 1d), the C_60_^‐^ and Zn^‐^ ions can be assigned to the C_60_‐SAM and ZnO, respectively. The peak intensity of the C_60_^−^ ions is increased at the surface of the ZnO in all samples, which indicate that the major amount of C_60_‐SAM molecules are at the surface of the ZnO. Meanwhile, the C_60_^−^ intensities in BHJ area were slightly increased in the BHJ:C_60_‐SAM films compared with the neat BHJ films, which indicates that a small amount of C_60_‐SAM remained in the BHJ layers. We note that a smaller amount of apparent C_60_‐ ions can be also detected in the BHJ films without C_60_‐SAM because of the long carbon backbones and side chains of the photoactive materials. Furthermore, to prove the formation of the ester group between ZnO and C_60_‐SAM, we conducted an X‐ray photoelectron spectroscopy (XPS) analysis on C_60_‐SAM, ZnO, and ZnO/C_60_‐SAM films. In the O *1s* core level spectra, we observed a blue‐shift in the binding energy of oxygen at the ZnO/C_60_‐SAM interface relative to that of ZnO film alone, implying the formation of chemical bonds at the ZnO/C_60_‐SAM interfaces (Figure S1a, Supporting Information). In Figure S1b,c (Supporting Information), the C *1s* core level spectra confirm the presence of an ester group at the interface. In Figure 1e, the absorption spectra of the D18, Y6, C_60_‐SAM, D18:Y6, and D18:Y6:C_60_‐SAM films were measured by UV–Vis–IR spectroscopy. Ground state absorption spectra of neat D18 and Y6 films showed maximum absorbance at 540 and 840 nm, respectively. Compared with the D18:Y6 film, the ternary blend film with 2 wt% of C_60_‐SAM did not exhibit any obvious change, consistent with the C_60_‐SAM showing negligible absorption in the corresponding region.

**Figure 2:**
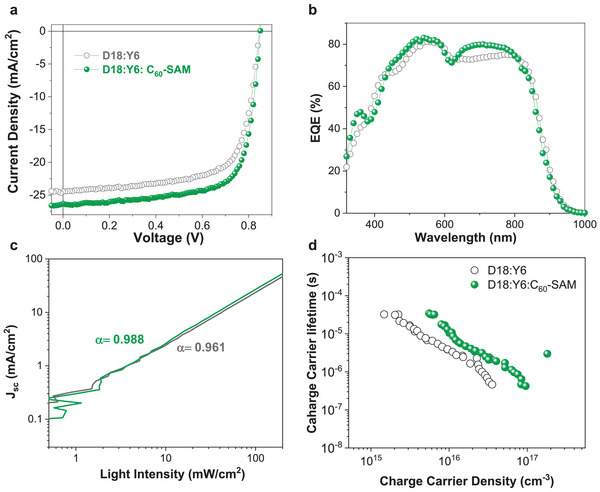
a) *J–V* curves, b) EQE spectra, c) light intensity dependent *J*_sc_ values, and d) charge carrier lifetime verses charge carrier density of organic photovoltaics (OPV) devices based D18:Y6 and D18:Y6:C_60_‐SAM systems.

To explore the impact of C_60_‐SAM in the BHJs on OPV performance, OPVs based on D18:Y6 and D18:Y6:C_60_‐SAM systems were fabricated with the structure described in Figure 1a. To find the best performance, we fabricated OPVs with different amount of C_60_‐SAM from 0 to 5 wt% in the BHJs as displayed in Figure S3 and Table S1 (Supporting Information). The ternary OPVs of D18:Y6 with 2 wt% of C_60_‐SAM showed optimal device performance, so we fixed on this condition for further analysis. Meanwhile, the OPVs with 5 wt% of C_60_‐SAM show significant decrease of *J*_sc_ and FF, and a slight change of *V*_oc_. Optical microscope images displayed in Figure S4 (Supporting Information) reveal a significant number of micron‐scale aggregates in the D18:Y6:C_60_‐SAM (5 wt%) film. We suspect that this suboptimal surface condition at the D18:Y6:C_60_‐SAM (5 wt%) film negatively impacts to the device performance. The current density voltage (*J*‐*V*) characteristics and the EQE spectra of D18:Y6 OPV devices with and without 2 wt% of C_60_‐SAM are shown in **Figures** **2**a,b, with the corresponding photovoltaic parameters summarized in **Table** **1**. We note that each OSC parameter described in the Table 1 is the average from 10 individual devices. The reference OPV without C_60_‐SAM exhibited the PCE of 14.4%, *V*_oc_ of 0.81 V, a *J*_sc_ of 25.0 mA cm^−2^, and a FF of 0.71, which are comparable to the reported literature values. In comparison with the reference devices, D18:Y6:C_60_‐SAM‐based ternary OPVs show the enhanced PCE of 15.0%, with *V*_oc_ of 0.81 V, a *J*_sc_ of 25.9 mA cm^−2^, and a FF of 0.72. It is apparent that the addition of 2 wt% C_60_‐SAM results in slight PCE improvement compared with reference cell, mainly due to the increase of *J*_sc_ and FF. The same effect was observed shown in the OPVs using a preformed ZnO/C_60_‐SAM bilayer as shown in Figure S5 and Table S2 (Supporting Information), with increases of the *J*_sc_ and FF, consistent with vertical separation of D18:Y6 and C_60_‐SAM layers. Moreover, dark *J*‐*V* characteristics in Figure S7 (Supporting Information) showed overall improved diode performance and lower reverse current for the devices with C_60_‐SAM compared to the reference device. These data clearly demonstrate an improvement in both *J*_sc_ and FF with the C_60_‐SAM, whereas the *V*_oc_ being invariant.

**Table 1:** The optimized photovoltaic performances of the organic photovoltaics (OPVs) based on D18:Y6 and D18:Y6:C_60_‐SAM under the illumination of AM 1.5G, 100 mW cm^−2^

BHJ	*V*_OC_ [V]	*J*_SC_ [mA cm^−2^] (cal. *J_sc_*)^a)^	FF	PCE [%]) (PCE_max_)
D18:Y6	0.81 ± 0.01	25.0 ± 0.68 (23.6)	0.71 ± 0.02	14.4 ± 0.34 (14.9)
D18:Y6:C_60_‐SAM	0.81 ± 0.02	25.9 ± 0.84 (24.5)	0.72 ± 0.02	15.0 ± 0.55 (15.6)

^Average values are obtained from 10 individual devices^

^a)^ The cal. *J*_sc_ values are obtained by integrating the EQE spectra.

We focus now on the increases in *J*_sc_ and FF with addition of C_60_‐SAM, which is well matched with the improved EQE curves as shown in Figure 2b. Because the absorption spectra of the D18:Y6 and D18:Y6:C_60_‐SAM films are not significantly changed (Figure 1e), we can conclude that the increases of the *J*_sc_ and FF are not contributed by the absorption of C_60_‐SAM or the BHJ. Next, steady state photoluminescence (PL) quenching measurements of D18:Y6 and D18:Y6:C_60_‐SAM films were carried out to confirm if the C_60_‐SAM affects the efficiency of charge separation at the BHJ interfaces. The relationship among PL quenching, morphology, and exciton transfer in OPVs is crucial for their overall efficiency. Optimal morphology ensures efficient exciton transfer and charge separation, leading to effective PL quenching. Based on the PL spectra presented in Figure S6 (Supporting Information), we can confirm that quenching occurred for both D18:Y6 and D18:Y6:C_60_‐SAM films by Y6 (excited at 550 nm). This suggests that C_60_‐SAM did not affect exciton separation in the D18. Furthermore, we observed excellent quenching for both films by D18 (excited at 780 nm), indicating that both films have efficient Y6 exciton separation. This is indicative of favorable film morphology for exciton separation in both devices and suggests that the difference in *J*_SC_ is not due to a difference in exciton separation.

To investigate this further, we performed light‐intensity (*P*_light_) dependent *J*_sc_ measurements to investigate possible bimolecular charge recombination losses during extraction. In general, the relationship of *J*_sc_ and *P*_light_ can be described as $J_{sc}\propto P_{\mathrm{light}}^{\alpha }$.^[^ ^32^, ^33^ ^]^ If the bimolecular recombination for the charge carriers in the OPVs is negligible, the power‐law index (*α*) will approach 1. As shown in Figure 2c, the *α* values of the devices based on D18:Y6 and D18:Y6:C_60_‐SAM systems were calculated to be 0.961 and 0.988, respectively. The slightly higher value of the ternary OPVs indicates that the suppressed bimolecular recombination, in agreement with the higher FF than the reference OPV. Moreover, charge‐recombination lifetime and drift mobility for these OPVs were determined from transient photovoltage (TPV) and charge extraction (CE) measurements (see Experimental Section for details).^[^ ^34^ ^]^ Figure 2d shows charge carrier lifetime as a function of carrier density obtained from TPV measurement demonstrate that lifetime at 2.3 × 10^16^ cm^−3^ carrier density (around 1 sun illumination) for D18:Y6 is 1.54 µs whereas for D18:Y6:C_60_‐SAM device it become 3.25 µs, which is almost twice from the control device. In Figure S7d (Supporting Information) higher drift‐mobility compared with reference OPVs above 1 sun illumination intensity due to fast de‐trapping/extraction of charge carriers. Also the fast CE under short‐circuit condition can be observed in Figure S7c (Supporting Information). Which is consistent with the higher value of *α*, and indicative of suppressed bimolecular recombination losses during charge transport and collection. Overall from CE–TPV analysis we can conclude that the enhanced *J_SC_* and FF observed the D18:Y6:C_60_‐SAM devices results from more efficient charge collection due to enhanced charge mobility and suppressed charge recombination.

**Figure 3:**
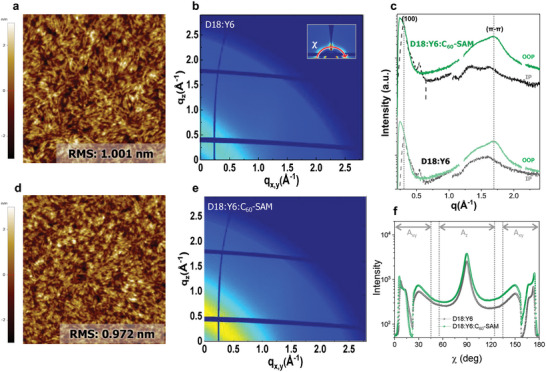
a) Atomic force microscopy (AFM) topography (2 × 2 µm) image and b) Grazing incidence wide‐angle X‐ray scattering (GIWAXS) pattern of D18:Y6 film. The inset of (b) is the polar angle (***χ***) range. c) In‐plane (black) and out‐of‐plane (green) line‐cut profiles of the D18:Y6 and D18:Y6:C_60_‐SAM films. d) AFM topography image and e) GIWAXS pattern of D18:Y6:C_60_‐SAM film. f) Pole figure analysis of the blend films extracted from the lamellar diffraction (100).

To investigate the morphological origin of the more efficient charge collection for the D18:Y6:C_60_‐SAM device, atomic force microscopy (AFM) and GIWAXS measurements were performed (**Figure** **3**a–f). AFM topography images of D18:Y6 (Figure 3a) and D18:Y6:C_60_‐SAM (Figure 3d) films showed that root‐mean square values of 1.00 and 0.97 nm, respectively, indicating that adding of C_60_‐SAM in the D18:Y6 system had no significant effect on the topography and surface roughness. Similar nanomorphologies were found in phase images of corresponding films (Figure S8, Supporting Information), which are consistent with the PL quenching data. Next, GIWAXS measurements were carried out to obtain a deeper insight into the molecular packing information. Prior to analysing the BHJ films, neat D18, Y6, and C_60_‐SAM films were investigated (Figure S9, Supporting Information). A line cut of neat D18 film exhibits a lamellar diffraction peak of 0.31 Å^−1^ (d spacing: 2.0 nm) and a *π*–*π* stacking diffraction peak of 1.6 Å^−1^ (*d* spacing: 0.39 nm), in agreement with previous literature.^[^ ^35^ ^]^ As shown in the Figure 3b,e, the (100) lamellar diffraction peaks appeared at *q*_xy_ and *q*_z_ directions in D18:Y6 and D18:Y6:C_60_‐SAM films, which indicate that face‐on and edge‐on orientations are coexisting in these films. Figure 3c displayed line‐cut profiles of D18:Y6 and D18:Y6:C_60_‐SAM films with the out‐of‐plane and in‐plane direction. The D18:Y6 film showed a (010) *π*–*π* stacking diffraction peak at 1.68 Å^−1^ (*d* spacing: 0.37 nm) and (100) diffraction peak at 0.31 Å^−1^ (*d* spacing: 2.0 nm). Similarly, the D18:Y6:C_60_‐SAM film exhibited a (010) *π*–*π* stacking diffraction peak at 1.68 Å^−1^ (*d* spacing: 0.37 nm) and (100) diffraction peak at 0.30 Å^−1^ (*d* spacing: 2.1 nm), indicating that the molecular characteristics are not significantly changed. Finally, to focus on molecular orientation, we performed a pole figure analysis of the blend films. Figure 3f displays the pole plot extracted from the (100) lamellar diffraction of the D18:Y6 and D18:Y6:C_60_‐SAM films. The inset of the Figure 3b is a close‐up of the lamellar diffraction over a range of the polar angles (*χ*). The lamellar diffraction peaks are extracted from the line cut data along the azimuthal angle from 0° to 180°. The integrated areas in the range of *χ* of 0–45° and 135–180° (*A*_xy_) and 55–125° (*A*_z_) were defined as face‐on and edge‐on orientation, respectively.^[^ ^36^, ^37^ ^]^ The fraction of face‐on to edge‐on ratio (*A*_xy_/*A*_z_) increased from 80% to 84% when the 2 wt% of C_60_‐SAM is added to D18:Y6, suggesting that the D18:Y6:C_60_‐SAM film prefers a more face‐on orientation than reference film. This enhanced face‐on preference in ternary blend is consistent with the slightly improved charge mobility and charge collections efficiency discussed above.

The results discussed above have shown that the C_60_‐SAM is able to affect the molecular orientation of the D18:Y6 blend and thus improve the device performance. To investigate the universality of this impact of C_60_‐SAM inclusion, OPVs with the different BHJ blends PTQ10:Y6 and PM6:Y6 with 1 wt% of C_60_‐SAM additive were fabricated (Figures S10 and S11, Supporting Information). Photovoltaic parameters of the OPVs and the GIWAXS pole plots extracted from the (100) lamellar diffraction data of the PTQ10:Y6 and PM6:Y6 systems are displayed in Table S3 and Figure S12 (Supporting Information), respectively. We note that solvent additives such as 1‐chloronaphthalene, which are commonly used in optimized PTQ10:Y6 and PM:Y6 OPVs, were not used to clearly see the effect of the C_60_‐SAM. We found similar C_60_‐SAM on both device efficiency and molecular orientation for both PTQ10:Y6 and PM6:Y6 systems. An improvement of *J*_sc_ and FF and therefore PCE in all three ternary devices was observed, while the *A*_xy_/*A*_z_ values of the ternary films increased. This results indicates that the C_60_‐SAM ternary system can be a universal strategy for improving OPV performances.

**Figure 4:**
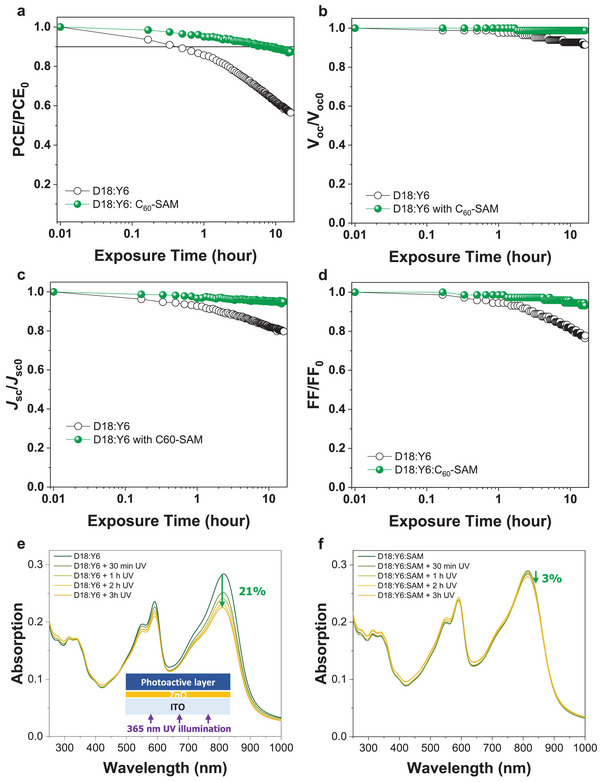
a) Normalized power conversion efficiency (PCE), b) *V*_oc_, c) *J*_sc_, and d) fill factor (FF) plotted against exposure time under 1 Sun illumination of D18:Y6 and D18:Y6:C_60_‐SAM devices in N_2_ condition. Absorption spectra of the e) D18:Y6 and f) D18:Y6:C_60_‐SAM films measured against exposure time under 365 nm UV light in N_2_ condition.

We now evaluate the photostability of the OPVs based on D18:Y6 and D18:Y6:C_60_‐SAM. The device operational stabilities of the devices without encapsulation were tested under 1 sun illumination in N_2_. Normalized PCE, *V*_oc_, *J*_sc_, and FF data are shown in **Figure** **4**a–d. Notably, the photostability of the device with the addition of 2 wt% of C_60_‐SAM is improved which retain 90% of their original PCE (*T*_90%_) after 8 h illumination, whereas the *T*_90%_ of theD18:Y6 device was only 0.33 h governed by *J*_sc_ and FF losses. In order to understand the improvement of photostability, we fabricated films with the structure of ITO/ZnO/D18:Y6 and ITO/ZnO/D18:Y6:C_60_‐SAM. Then, UV–Vis absorption spectra of the films were measured before and after 365 nm UV illumination in N_2_ condition for 3 h. In Figure 4e,f, we found a 11% and 21% loss of absorption from their initial intensities at the peaks of 590 and 815 nm, respectively, in D18:Y6 film. These peaks at 590 and 815 nm result from D18 and Y6, respectively; these results suggest that decomposition of Y6 is more severe than that of D18. In contrast, the D18:Y6:C_60_‐SAM ternary film showed only small spectral changes of 0% and 3% from their initial values at 590 and 815 nm, respectively. We also carried out the UV stability test on the PTQ10:Y6 and PM6:Y6 system as displayed in Figure S13 (Supporting Information). As expected, the absorption spectra of PTQ10:Y6 and PM6:Y6 against UV illumination showed similar tendency with D18:Y6 system, which is that the Y6 peak is significantly bleached, whereas the ternary system successfully suppressed the photoinduced absorption bleaching. From this improvement of photostability against 1 sun and UV light, we can expect that the effective passivation of UV driven ZnO photocatalytic reactions by the C_60_‐SAM.

## 3 Conclusion

In summary, we have successfully demonstrated that device performance and photostability can be improved by using a ternary blend with C_60_‐SAM. The C_60_‐SAM in the ternary system encourages a more favorable molecular orientation of BHJ from edge‐on to face‐on orientation, correlated with an improvement in charge carrier lifetime and mobility, thereby resulting in the increases in *J*_sc_ and FF for the ternary OPVs. In addition, the vertically phase separated C_60_‐SAM successfully protects the BHJ layer from the UV light by passivating of the ZnO surface, leading to a significant improvement of photostability. By introducing this ternary strategy to other BHJ blends such as PTQ10:Y6 and PM6:Y6, we demonstrated the universality of this vertically self‐assembled C_60_‐SAM approach. Therefore, these results give a new and simple approach to increasing both photostability and efficiency in inverted OPVs.

## 4 Experimental Section

### Materials

D18, PM6, PTQ10, Y6, and C_60_‐SAM were purchased from 1‐material.

*Solution Preparation*: For the ZnO solution, 219.5 mg of zinc acetate dehydrate precursor was dissolved in 2 mL of 2‐methoxyethanol with 60.4 µL 1‐ethanolamine. D18 and Y6 were dissolved in chloroform. The concentration for donor and acceptor combination (1:1.2 ratio by weight) were 8.8 mg mL^−1^. The photoactive solution was stirred for at least 6 h at 40 °C and cooled down before use. For the C_60_‐SAM solution, 5, 10, 20, and 50 mg of C_60_‐SAM were dissolved in 1 mL of IPA for 0.5, 1, 2, and 5 wt% solutions, respectively. The C_60_‐SAM solution was stirred overnight at 25 °C and then added to BHJ solution with 0.5 vol% ratio to make the BHJ:C_60_‐SAM solutions.

### Device Fabrication

The printed OSCs were fabricated with an inverted structure (Glass/ITO/ZnO/photoactive layer/MoO_3_/Ag). Before device fabrication, glass substrates with patterned ITO were treated sequentially in deionized‐water for 10 min, acetone for 20 min, and isopropyl alcohol for 10 min by ultrasonicator, followed by UV–ozone treatment for 30 min. ZnO film was deposited on the substrate by spin‐coating at 4000 rpm for 30s. The film was annealed at 180 °C for 20 min in air to form a 30 nm thick ZnO film. The D18:Y6 and D18:Y6:C_60_‐SAM photoactive layers were coated on the ZnO film by spin‐coating process. MoO_3_ (10 nm) and Ag (100 nm) layers were deposited by evaporation through a shadow mask yielding active areas of 0.045 cm^2^ in each device.

### Characterization

*J–V Characterization and IPCE*: The *J–V* characteristics of OSCs were measured using an Iviumsoft apparatus with simulated AM 1.5 illumination (100 mW cm^−2^) via a solar simulator (Abet Technologies Sun 3000) under normal atmospheric conditions. The IPCE spectra were obtained using a solar cell spectral response/QE/IPCE measurement system (Quant X‐300, Newport Corp.) calibrated with an 603 621 reference detector (Newport Corp.). The chopping frequency of IPCE measurement was 100 Hz. The cal. *J*_sc_ from EQE spectra was <6% mismatch with those measured by *J*–*V* measurements.

*AFM and GIWAXS Measurement*: AFM images of photoactive layers were obtained using XE‐100, Park systems, Inc. GIWAXS measurement were conducted at the 3C‐WAXSI beamline in the Pohang Accelerator Laboratory (PAL) using a monochromatized X‐ray radiation source of 10.55 eV (*λ* = 1.170 Å) and a 2D charge‐coupled device (CCD) detector (model Rayonix 2D SX 165, Rayonix, Evanston, IL, USA). The samples were mounted on a *z*‐axis goniometer equipped with a low vacuum chamber (≈10^−3^ Torr).

*Absorption and PL*: UV–Visible spectra of the thin films were acquired with a PerkinElmer Lambda 750 spectrometer in air. The PL spectra were measured with a Fluorolog‐3 spectrofluorometer (Horiba Jobin Yvon). The excitation wavelength was 780 nm.

*ToF–SIMS Measurement*: The ToF–SIMS experiments were conducted using TOF‐SIMS M6 (IONTOF GmbH) instrument equipped with a Ar+ sputter source with a 30 keV energy and 0.935 nA current. The typical sputter area was 15 by 15 mm and height ≈0.3 mm.

*Charge Extraction and Transient Photovoltage (CE/TPV)*: CE on solar cells was performed at open circuit condition under different light intensity upto 10 Sun provided by12 white LEDs connected to a remote‐controlled power supply. These LEDs turn on time is 100 ms to allow DUT to achieve steady state condition with switch off time is 100 ns. CE transient was recorded over 50 Ω resistance on TDS 3032 Tektronix digital oscilloscope connected to the computer via NI‐PCI‐6251 DAQ card. Simultaneously the current transient was integrated with respect to time to estimate carrier density. Carrier density under short circuit and open‐circuit condition depends on anode connection during measurement. TPV transient was recorded at open circuit condition under the equivalent illumination intensity to the CE measurement and fitted with a single exponential function to obtain a carrier lifetime. Reliability of fitting for TPV and CE measurement was confirmed by reconstructing *V*_oc_ (Figure S14, Supporting Information). Open circuit condition was confirmed by selecting 1 MΩ input impedance of oscilloscope. An Nd:YAG pulsed laser at 532 nm (Continuum Minilite II) with a pulse width of smaller than 10 nm and 5 Hz repletion rate was used to generate small perturbations in the device. The measurement of short‐circuit current and carrier density (*n*_sc_)under short circuit condition under different illumination intensity allows the calculation of drift mobility. Therefore, effective mobility µ* can be calculated with correction factor *f(δ) as* follows.

(1) $$\mu *=-\frac{J_{\mathrm{sc}}*d}{e*n_{\mathrm{sc}}}*\left(\frac{1}{f\left(\delta \right)*V_{\mathrm{int}}}\right)$$

where *d* is thickness of active layer, *e* is electronic charge, and *V*_int_ is built in voltage.

## Conflict of Interest

The authors declare no conflict of interest.

## Supporting information

Supporting Information

## Data Availability

The data that support the findings of this study are available from the corresponding author upon reasonable request.
