# Supplementary material for: New Ternary Blend Strategy Based on a Vertically Self‐Assembled Passivation Layer Enabling Efficient and Photostable Inverted Organic Solar Cells
Source: Adv Sci (Weinh). 2023 Apr 25;10(17):2206802. doi: 10.1002/advs.202206802 (PMC10265084; doi:10.1002/advs.202206802)
Supplement: Supplementary file 1 — Supporting Information [file ADVS-10-2206802-s001.pdf]

ADVANCED SCIENCE
Open Access

Supporting Information

for *Adv. Sci.*, DOI 10.1002/advs.202206802

New Ternary Blend Strategy Based on a Vertically Self-Assembled Passivation Layer Enabling Efficient and Photostable Inverted Organic Solar Cells

Soyeong Jeong, Aniket Rana, Ju-Hyeon Kim, Deping Qian, Kiyoung Park, Jun-Ho Jang, Joel Luke, Sooncheol Kwon, Jehan Kim, Pabitra Shakya Tuladhar, Ji-Seon Kim, Kwanghee Lee, James R. Durrant* and Hongkyu Kang**

Supporting Information

A New Ternary Blend Strategy Based on a Vertically Self-assembled Passivation Layer Enabling Efficient and Photo-stable Inverted Organic Solar Cells

Soyeong Jeong[a], Aniket Rana[a], Ju-Hyeon Kim[b], Deping Qian[a], Kiyoung Park[b], Jun-Ho Jang[c], Joel Luke[d], Sooncheol Kwon[e], Jehan Kim[f], Pabitra Shakya Tuladhar[a], Ji-Seon Kim[d], Kwanghee Lee[b,d,e,], James R. Durrant[a,]*, Hongkyu Kang[g,]**

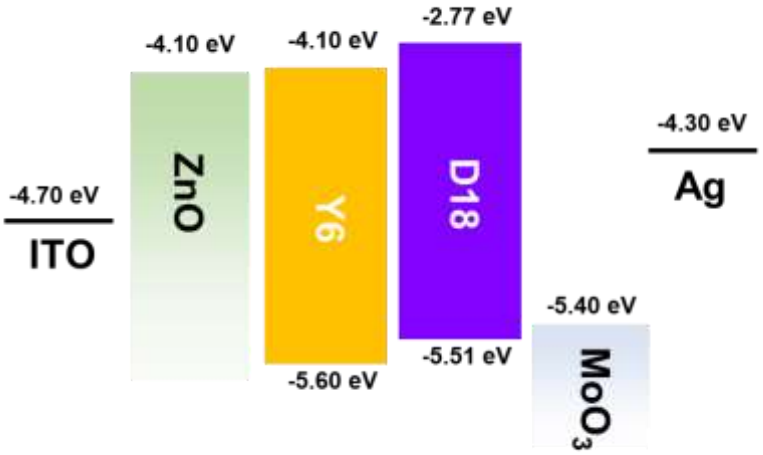

Figure S1 Energy levels of inverted OPV used in this study

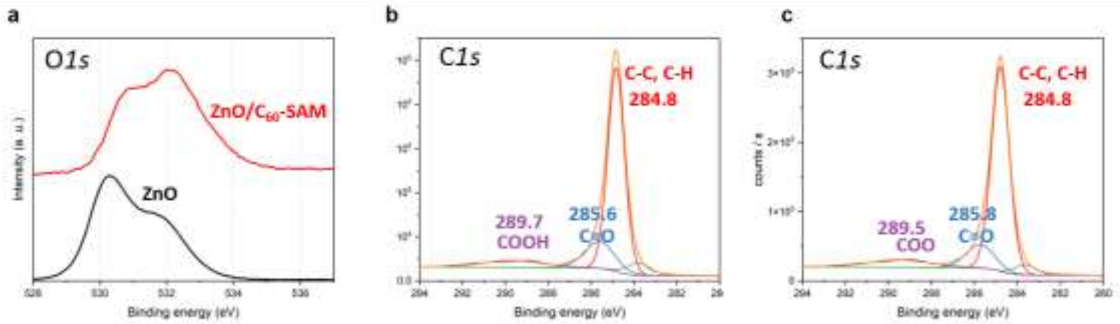

Figure S2 The X-ray photoelectron spectra of (a) O *1s* for the ZnO and ZnO/C_{60}-SAM, C *1s* for the (b) C_{60}-SAM, and (c) ZnO/C_{60}-SAM films deposited on silicon substrate.

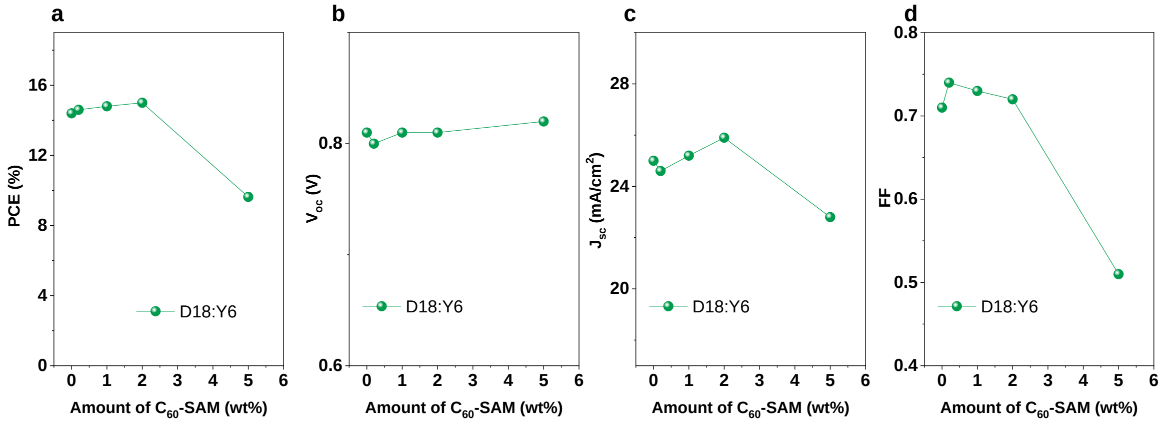

Figure S3 OPV parameters for D18:Y6 as a function of amount of C_{60}-SAM additive.

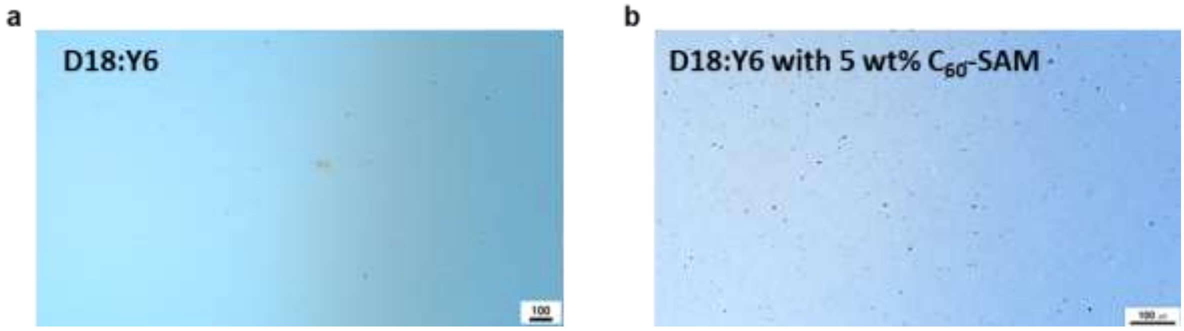

Figure S4 Optical microscope images of the a) D18:Y6 film, b) D18:Y6:C_{60}-SAM film with 5 wt% of C_{60}-SAM.

Table S1 The photovoltaic performances of the OPVs for D18:Y6 as a function of amount of C_{60}-SAM additive under the illumination of AM 1.5G, 100 mW cm^{-2}. Average values are obtained from 5 individual devices.

**Amount of C_{60}-SAM (wt%)**	**V_{OC} (V)**	**J_{SC} (mA cm^{-2})**	**FF**	**PCE (%)**
0	0.81±0.01	25.0±0.68	0.71±0.02	14.4±0.34
0.5	0.80±0.01	24.6±0.31	0.74±0.03	14.6±0.56
1	0.81±0.00	25.2±0.73	0.73±0.01	14.8±0.09
2	0.81±0.02	25.9±0.84	0.72±0.02	15.0±0.55
5	0.82±0.01	22.8±1.65	0.51±0.03	9.63±1.27

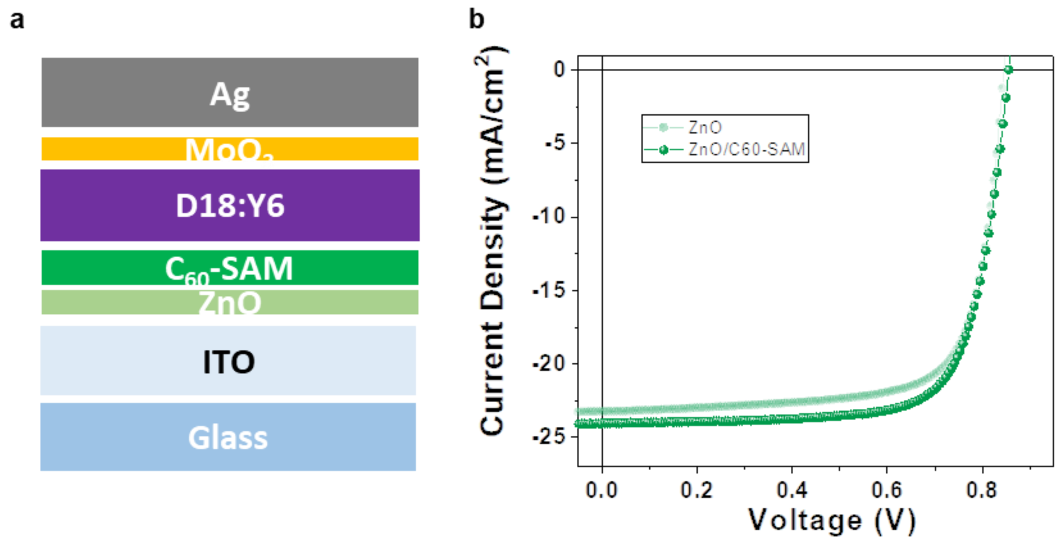

Figure S5 a) Device structure with the preformed ZnO/C_{60}-SAM bilayer interface before BHJ deposition . b) *J-V* curve of the OPVs without and with C_{60}-SAM interlayer.

Table S2 The photovoltaic performances of the D18:Y6-based OPVs without and with the C_{60}-SAM interlayer under the illumination of AM 1.5G, 100 mW cm^{-2}. Average values are obtained from 5 individual devices.

Active	ETL	V_{OC} (V)	J_{SC} (mA cm^{-2})	FF	PCE (%)
D18:Y6	ZnO	0.85±0.00	22.6±0.77	0.73±0.00	14.1±0.46
	ZnO/C_{60}-SAM	0.85±0.00	24.0±0.22	0.73±0.01	14.9±0.29

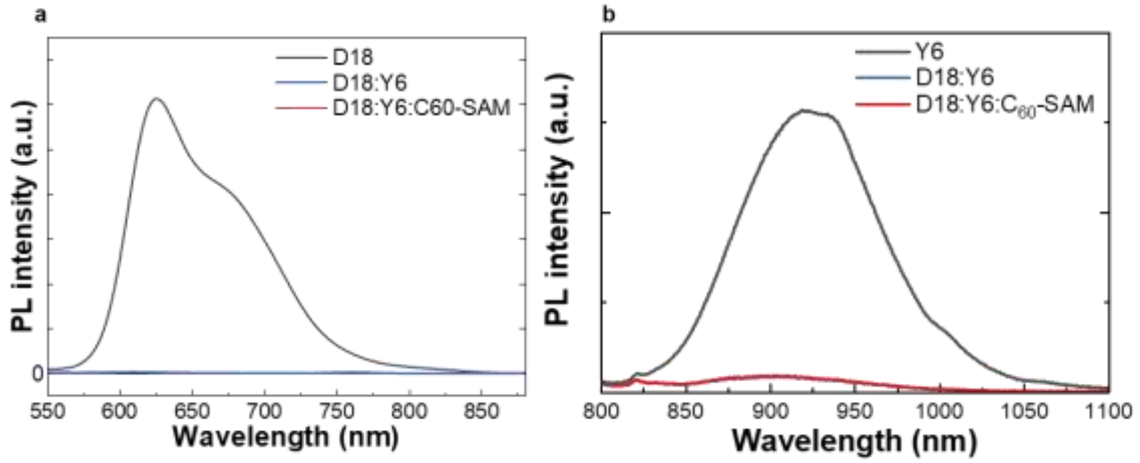

Figure S6 Steady-state photoluminescence spectra of a) D18, D18:Y6 and D18:Y6:C_{60}-SAM films (excitation wavelength = 550 nm) and b) Y6, D18:Y6 and D18:Y6:C_{60}-SAM (excitation wavelength = 780 nm).

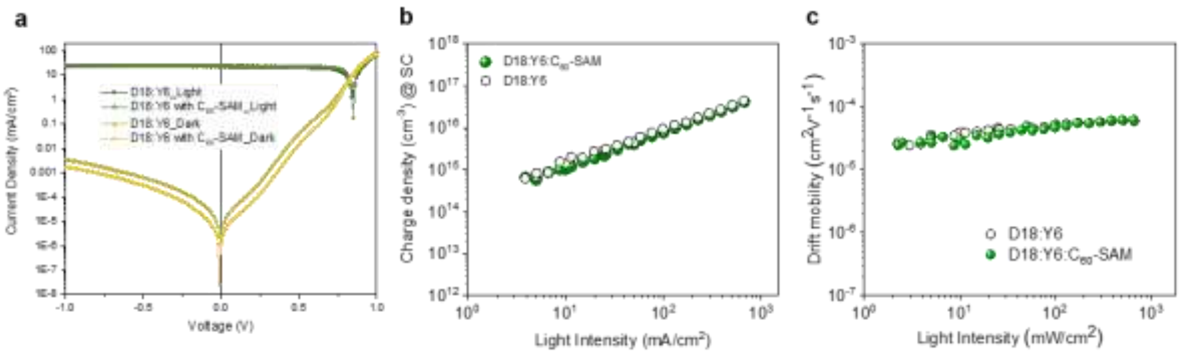

Figure S7 a) Rrepresentative light and dark *J-V* curves from D18:Y6 and D18:Y6:C_{60}-SAM OPVs. b) Charge density and c) drift mobility for the D18:Y6 and D18:Y6:C_{60}-SAM OPVs as a function of light intensity.

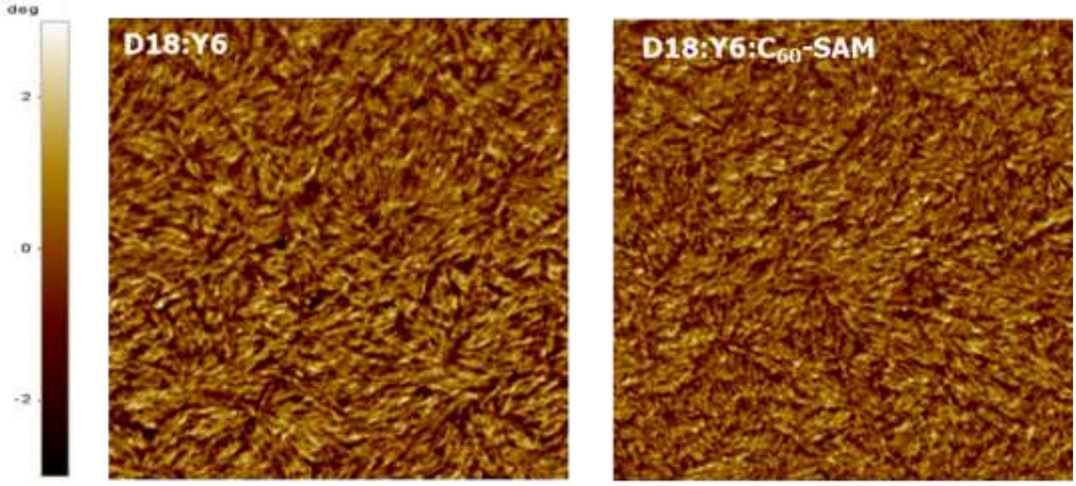

Figure S8 AFM phase images (2 μm × 2 μm) of the D18:Y6 and D18:Y6:C_{60}-SAM films.

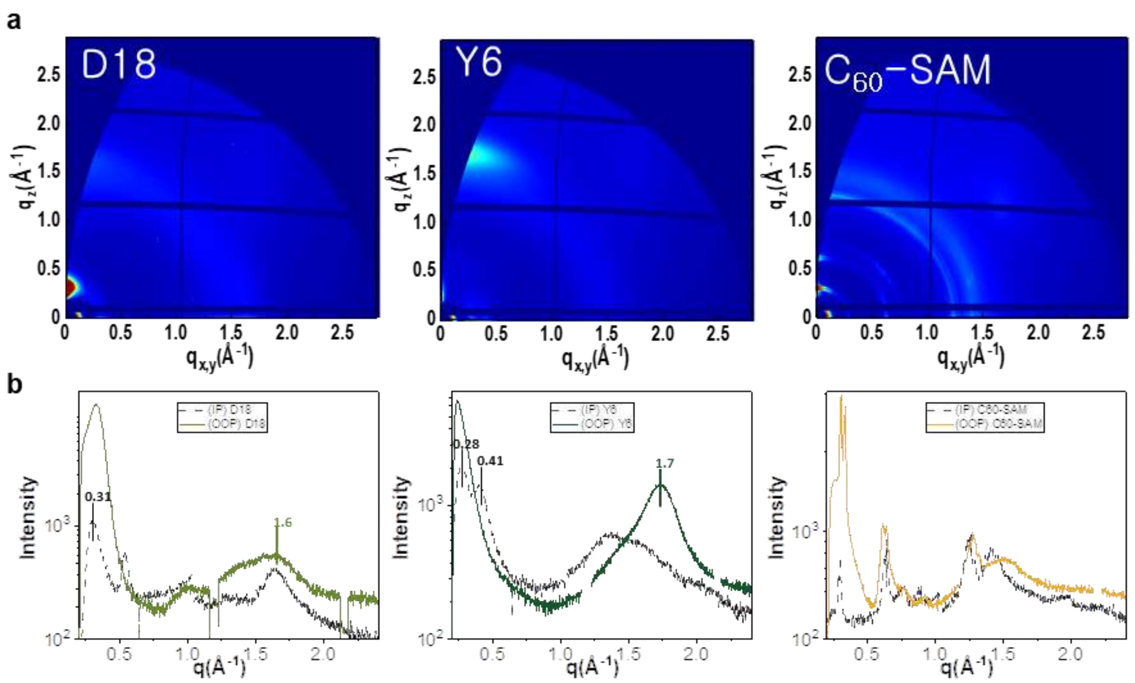

Figure S9 GIWAXS 2D scattering a) patterns and b) corresponding line-cuts of the neat films of the materials used in this study.

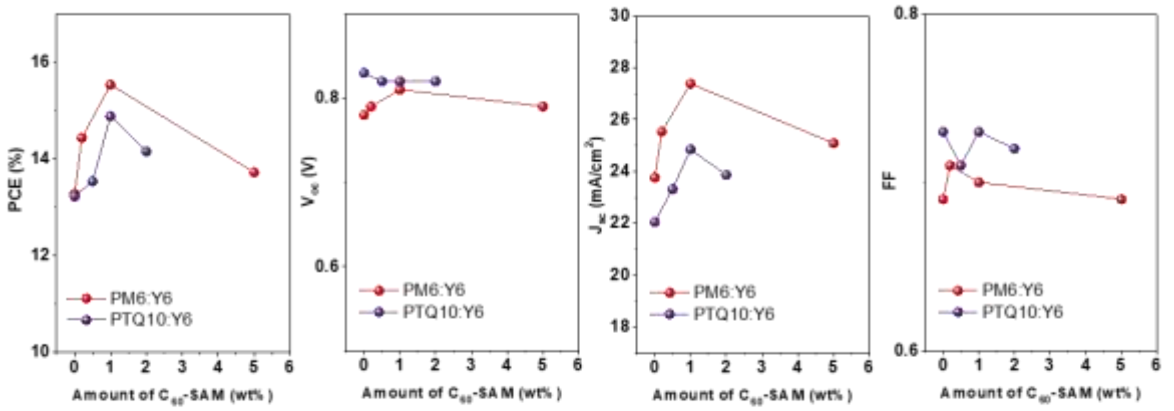

Figure S10 Device parameters for PM6:Y6 and PTQ10:Y6 as a function of amount of C_{60}-SAM.

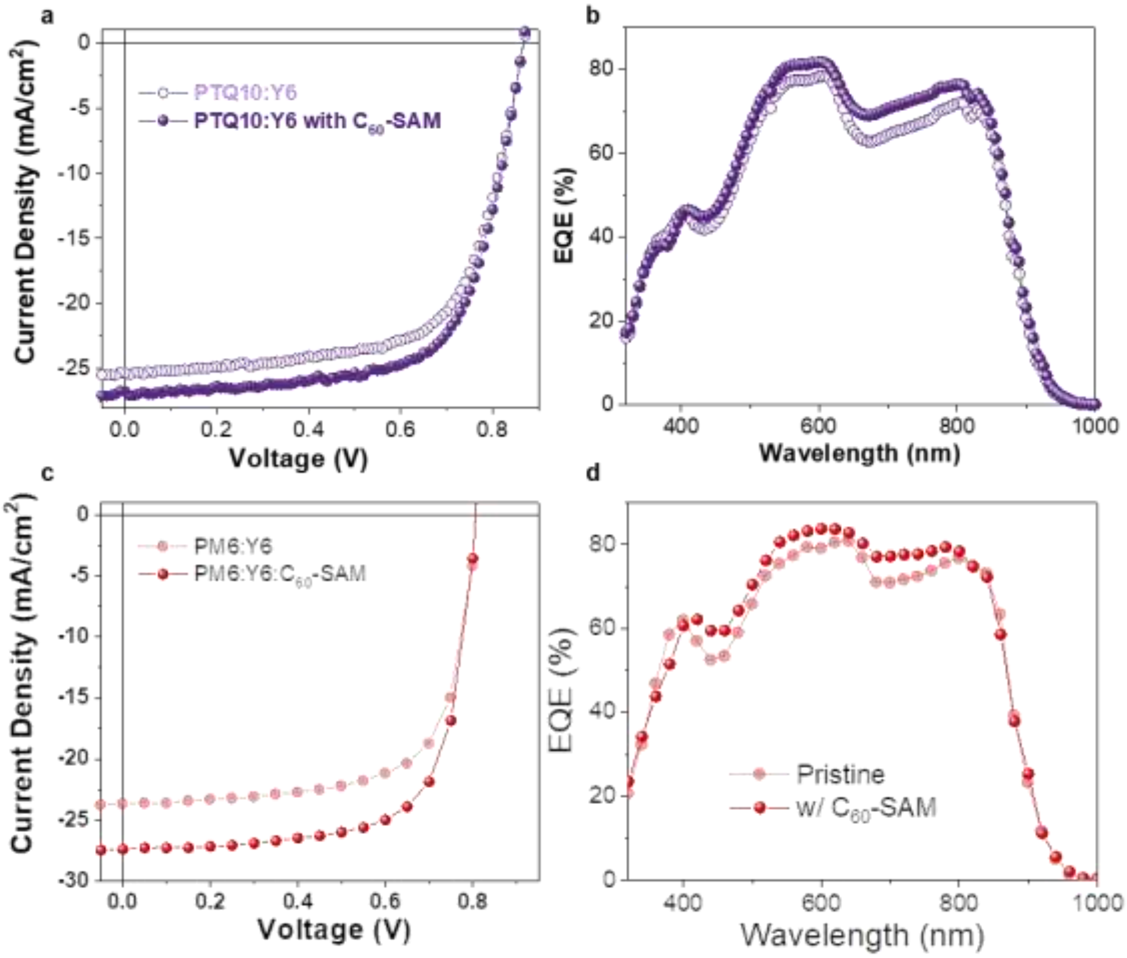

Figure S11 *J*–*V* curves and EQE spectra of OPV devices based on PTQ10:Y6 (a and b) and PM6:Y6 (c and d) systems.

Table S3 The optimized photovoltaic performances of the OPVs based on PTQ10:Y6 and PM6:Y6 systems under the illumination of AM 1.5G, 100 mW cm^{-2}. Average values are obtained from 5 individual devices.

**BHJ**	**V_{OC} (V)**	**J_{SC} (mA cm^{-2})**	**FF**	**PCE (%) (PCE_{max})**
PTQ10:Y6	0.86±0.02	25.34±0.74	0.67±0.01	14.64±0.76 (15.6)
PTQ10:Y6:C_{60}-SAM	0.86±0.03	26.42±1.17	0.68±0.03	15.19±0.64 (16.3)
PM6:Y6	0.81±0.00	23.82±0.85	0.68±0.02	13.06±0.29 (13.3)
PM6:Y6:C_{60}-SAM	0.81±0.01	25.40±1.62	0.70±0.01	14.31±0.91 (15.5)

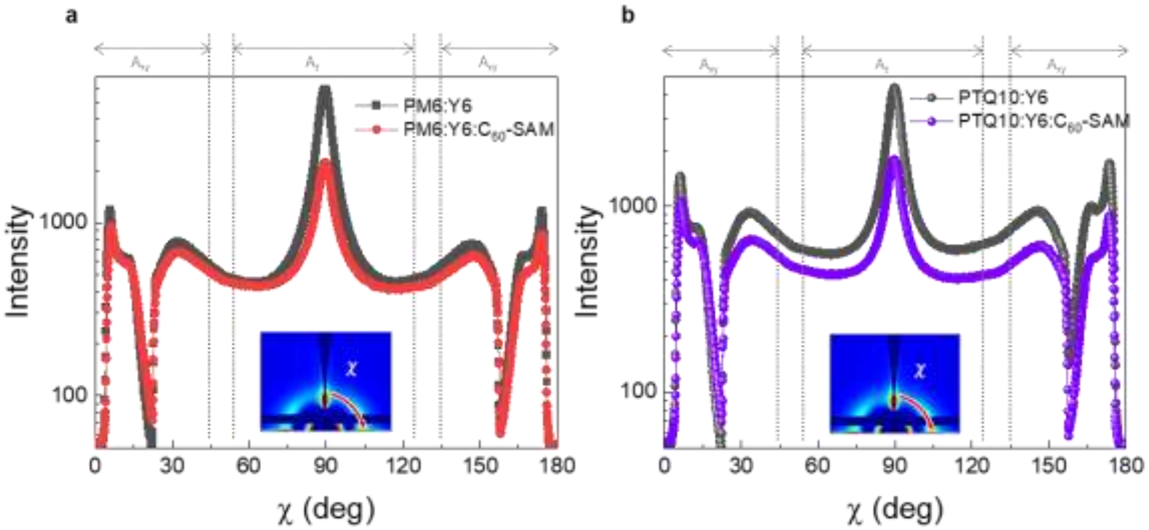

Figure S12 Pole figures analysis of the PM6:Y6 (a) and PTQ10:Y6 (b) based blend films extracted from the lamellar diffraction (100). The inset of (b) is the polar angle (χ) range.

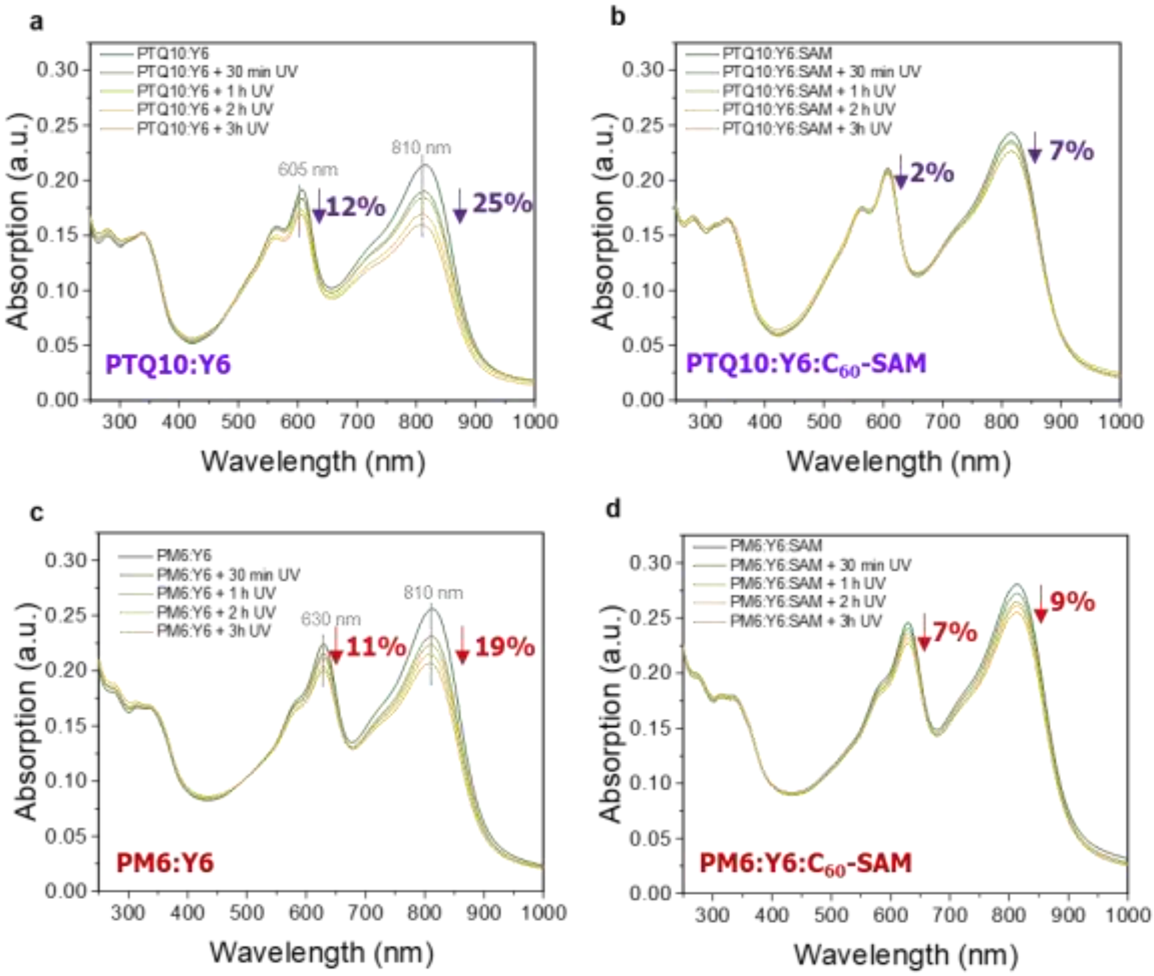

Figure S13 Absorption spectra of the (a,b) PTQ10:Y6 and (c,d) PM6:Y6 systems measured against exposure time under 365 nm UV light in N_2 condition.

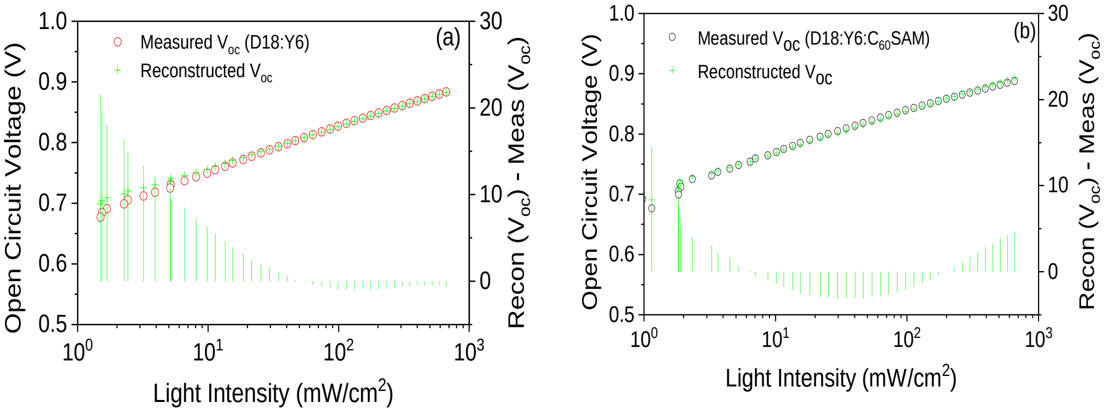

Figure S14 Light intensity dependent open-circuit voltage for (a) D18:Y6 (b) D18:Y6:C_{60}-SAM. Circle symbol indicate measured V_{oc} , and (+) symbol indicate reconstructed V_{oc} from TPV and CE measurements. Identical values of reconstructed *V*oc confirm the reliability of TPV and CE measurements.
